# Conventional and Specific-Pathogen Free Rats Respond Differently to Anesthesia and Surgical Trauma

**DOI:** 10.1038/s41598-019-45871-z

**Published:** 2019-06-28

**Authors:** Hayley L. Letson, Jodie Morris, Erik Biros, Geoffrey P. Dobson

**Affiliations:** Heart, Trauma and Sepsis Research Laboratory, College of Medicine and Dentistry, James Cook University, Queensland, 4811 Australia

**Keywords:** Cardiovascular biology, Experimental models of disease

## Abstract

Specific-pathogen free (SPF) animals were introduced in the 1960s to minimize disease and infection as variables in biomedical research. Our aim was to examine differences in physiological response in rat colonies bred and housed in a conventional versus SPF facility, and implications for research. Sprague-Dawley rats were anesthetized and catheterized for blood and pressure monitoring, and electrocardiogram (ECG) leads implanted. Hematology was assessed, and coagulation profile using rotational thromboelastometry. Health screening was outsourced to Cerberus Sciences. SPF rats had significantly lower pulse pressure (38% decrease), arrhythmias and prolonged QTc (27% increase) compared to conventional rats. No arrhythmias were found in conventional rats. SPF rats had significantly higher white cell, monocyte, neutrophil and lymphocyte counts, and were hyperfibrinolytic, indicated by EXTEM maximum lysis >15%. Independent assessment revealed similar pathogen exclusion between colonies, with the exception of *Proteus* in SPF animals. Returning to a conventional facility restored normal host physiology. We conclude that SPF animals displayed an abnormal hemodynamic, hematological and hemostatic phenotype in response to anesthesia and surgery, and provide a number of recommendations to help standardize research outcomes and translation.

## Introduction

In the 1950s researchers were increasingly frustrated with the presence of disease or infection as an unwanted variable affecting their experiments^1^. As a result of an international mandate for better quality animals^2^, the National Institutes of Health (NIH) issued guidelines for animal housing and husbandry in 1962^3^. Since that time a great deal of attention has been paid to standardizing facilities, light/day cycles, diets, techniques and equipment^4^. The overall strategy has been to breed animals free of infectious agents, from germ-free stock or caesarean aseptic techniques, then expose the colony to an environment free of selected *infectious* pathogens (not all) that would otherwise interfere with research objectives^2,4^. Today, most animal husbandry facilities adopt this specific-pathogen free (SPF) approach and provide certification that a colony or breeding pair is free from common infectious agents that a species is customarily exposed to in the wild. This approach also has additional benefits of cost-saving and ethical benchmarks as it reduces the number of animals used within individual studies. It also aims to improve standardization of animals and the scale of reproducibility (and replicability) among different laboratories around the world working on similar problems and preclinical questions^1,4,5^.

However, over the past decade, there have been a number of anomalies creeping into the literature and growing concerns about the clinical relevance of SPF laboratory rodents. Recent studies have reported broad and unexpected changes in the immune system of SPF animals, which have been linked to changes in the gut microbiome^5,6^. Changes in the gut microbiome influences animal models for metabolic disorders, obesity, hematopoiesis, osteoarthritis, inflammatory bowel diseases, allergies, autoimmunity, cancer and neuropsychiatric diseases^7–10^. More recently, changes in the microbiome were shown to lead to numerous physiological disorders by altering an animal’s circadian rhythm^11^.

We report that animal breeding and housing changes at James Cook University from a conventional facility to a new SPF facility resulted in profound alterations in the physiology of Sprague Dawley rats, and their response to minor trauma. The physiological variables included decreased tolerance to anesthesia, hemodynamic instability, aberrant hematology, traumatic bleeding and reduced physiological reserve in SPF animals. This altered phenotype to the stress of surgical trauma was completely reversed when animals were returned to the original conventional facility.

## Results

### Hemodynamics and electrocardiograph

**Figure 1:**
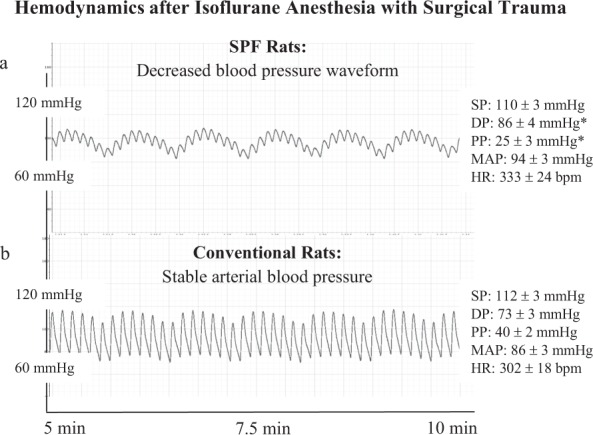
Representative blood pressure traces from SPF (**a**) and Conventional (**b**) male Sprague Dawley rats taken under isoflurane anesthesia following surgical instrumentation. Values represent mean ± SEM of n = 5 rats from each colony. SP = systolic pressure; DP = diastolic pressure; PP = pulse pressure calculated from SP – DP; MAP = mean arterial pressure; HR = heart rate. *p < 0.05 compared to Conventional rats. Power (1-β err prob) = 0.97 (Critical t = 1.86, Df = 8, α = 0.05, outcome measure = PP).

**Figure 2:**
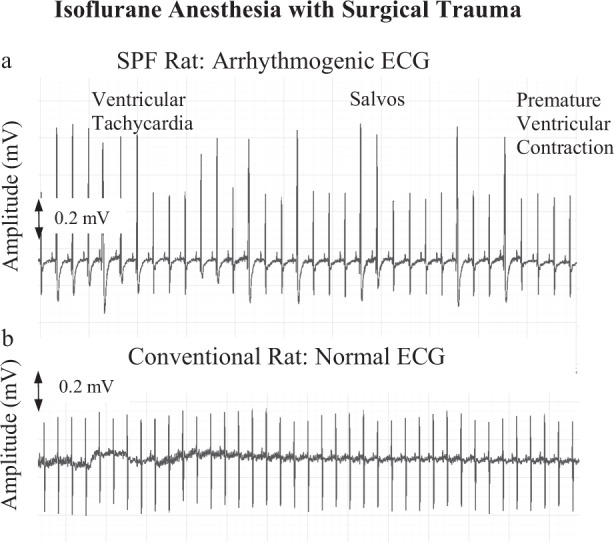
Representative lead II electrocardiograms (ECG) from SPF (**a**) and Conventional (**b**) male Sprague Dawley rats taken under isoflurane anesthesia following surgical instrumentation (n = 20 each group). SPF rats were arrhythmogenic with incidences of ventricular tachycardia, Salvos, bigeminy, and premature ventricular contractions. See Table 1 for data.

Arterial blood pressure trace in spontaneous breathing SPF animals showed significantly reduced pulse pressure (systolic pressure minus diastolic pressure) despite similar mean arterial pressure (MAP) and heart rate (HR) compared to conventional rats (Fig. 1a,b). Timing of oscillations in the blood pressure waveform during spontaneous breathing were similar between the two colonies. The ECG of SPF animals showed a range of abnormal cardiac rhythms including premature ventricular contractions (skipped beats), salvos and bigeminy episodes (Fig. 2a,b). Arrhythmias were found in 85% of SPF animals (17/20) compared to no arrhythmias in the conventional rats (*p* < 0.001) (Table 1). There were no differences in RR or QRS complex duration, however SPF rats had significantly prolonged QTc intervals (0.14 vs. 0.11 sec; *p* = 0.033) (Table 1).

**Table 1:** Episodes of arrhythmias and ECG parameters of male Sprague-Dawley Conventional and SPF rats after 7 days acclimation in the laboratory prior to experimentation.

Parameter	Conventional (n = 20)	SPF (n = 20)	*p* value
Total Arrhythmias	0 ± 0	21.55 ± 5.91 (0–87)	< 0.001
PVCs	0 ± 0	16.15 ± 4.48 (0–74)	< 0.001
Salvos	0 ± 0	3.10 ± 1.40 (0–25)	0.014
Bigeminy	0 ± 0	1.65 ± 0.60 (0–8)	0.014
VT	0 ± 0	0.65 ± 0.33 (0–4)	0.289
RR (sec)	0.20 ± 0.01	0.19 ± 0.01	0.458
QRS (sec)	0.02 ± 0.00	0.02 ± 0.00	0.994
QT (sec)	0.05 ± 0.00	0.06 ± 0.01	0.114
QTc (sec)	0.11 ± 0.01	0.14 ± 0.01	0.033

Values are mean ± SEM with range in parentheses. Episodes of arrhythmias were counted over a 20-min period following surgical instrumentation. SPF = specific-pathogen free; PVCs = premature ventricular contractions; VT = ventricular tachycardia; QTc = Bazett-corrected QT interval. Power (1-β err prob) = 1.00 (Critical t = 1.69, Df = 38, α = 0.05, outcome measure = total arrhythmias).

### Coagulation profile

**Figure 3:**
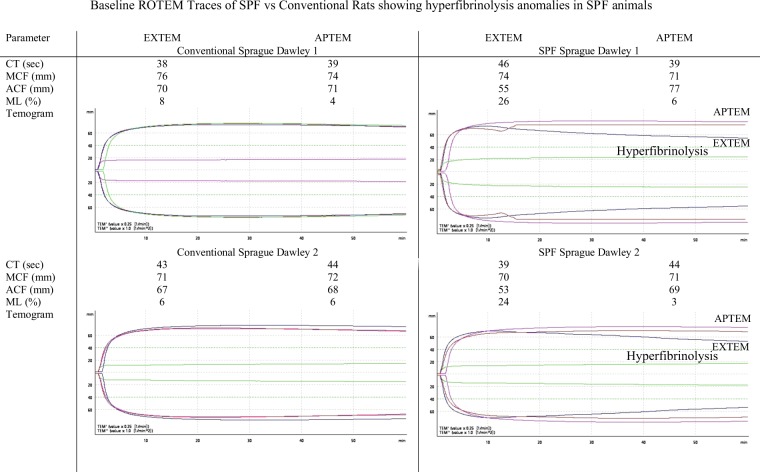
ROTEM parameters and Temograms from n = 2 Conventional and n = 2 SPF male Sprague Dawley rats demonstrating hyperfibrinolysis in SPF rats. EXTEM = extrinsically-activated test using tissue factor; APTEM = activation as for EXTEM with aprotinin; CT = clot time; MCF = maximum clot firmness; ACF = actual clot firmness (clot amplitude 60 min following clot initiation); ML = maximum lysis.

ROTEM analysis showed no differences in EXTEM clot times or clot amplitude (maximum clot firmness) between SPF and conventional rats. However, both SPF animals were hyperfibrinolytic, indicated by EXTEM maximum lysis (ML) percentages >15 (26% and 24% vs. 8% and 6% in conventional rats) (Fig. 3). Hyperfibrinolysis in SPF rats was confirmed with APTEM test which showed correction of ML to 6% and 3% with the addition of aprotonin which inhibits plasmin activation of fibrinolysis.

### Blood counts

The baseline total blood counts for conventional and SPF rats is shown in Table 2. The data indicate that the SPF rats had an overall aberrant hematology, a lower haemoglobin level and a 1.5-fold increase in platelet count compared to conventional rats. The number of white cells and lymphocytes were 1.4-fold higher than in conventional rats (*p* < 0.05), and monocyte counts 2.7-fold higher. Neutrophils were also 1.6-fold higher in SPF rats (*p* < 0.05). The hematological data of Said and Abiola^12^ highlight the differences in the SPF and conventional animals, supporting the notion of an aberrant hematology (Table 2).

**Table 2:** Baseline blood counts for male Sprague Dawley Rats.

Parameter	Conventional (n = 100)	JCU SPF (n = 34)	*Said and Abiola (12)
WBC (x10^9^/l)	9.62 ± 0.25	13.29 ± 0.86*	9.04 ± 2.29
LYM (x10^9^/l)	7.01 ± 0.17	9.57 ± 0.85*	7.50 ± 1.91
MON (x10^9^/l)	0.51 ± 0.03	1.39 ± 0.47	0.55 ± 0.25
NEU (x10^9^/l)	2.09 ± 0.10	3.25 ± 0.33*	NR
RBC (x10^12^/l)	7.17 ± 0.07	7.36 ± 0.22*	7.61 ± 0.27
PLT (10^9^/l)	168 ± 16	247 ± 33*	NR
HgB (g/dL)	15.58 ± 0.14	13.91 ± 0.45*	15.63 ± 0.71
HCT (%)	38.17 ± 0.40	39.82 ± 1.41*	NR

Values are mean ± SEM with number of animals in parentheses. *p < 0.05 compared to JCU conventional colony. Power (1-β err prob) = 0.999 (Critical t = 1.66, Df = 132, α = 0.05, outcome measure = WBC). SPF = specific-pathogen free; NR = not reported; WBC = white blood cells; LYM = lymphocytes; MON = monocytes; NEU = neutrophils; RBC = red blood cells; HgB = hemoglobin; HCT = hematocrit; PLT = platelets. Data from conventional animals were from a trauma study carried out before transition to SPF animals (see Materials and Methods). Said & Abiola (2014) housed male SD rats (117–264 g) in RC1 propylene cages with metal grill tops (conventional) and collected whole blood for hematological analysis following cervical dislocation and decapitation.

*Said and Abiola^12^.

### Health screening

The externally commissioned health screening for randomly selected rats from the SPF, conventional and SPF animals acclimated for 7 days in the laboratory are shown in Table 3. The standout result is the presence of *Proteu*s spp. in the two SPF groups and not the conventional rats. No animals had pinworms. One out of two animals from all groups had the presence of *Entamoeba muris*. One SPF animal had *Staphylococcus aureus* present, however this bacterium was not found in the 7-day laboratory acclimated SPF or conventional rats (Table 3). Viral screening using enzyme immunoassay (EIA) and indirect fluorescent antibody (IFA) techniques confirmed all rats were negative for Kilham Rat virus, Parvovirus (rNS1), Pneumonia Virus of Mice, Rat Coronavirus, Rat Minute Virus, Rat Parvovirus Rat Theilovirus, and Toolans H-1.

**Table 3:** Fecal microbiology of male Sprague-Dawley Conventional, SPF rats and SPF rats after 7 days acclimation in the laboratory prior to experimentation.

Bacteria and Fungi	Test Method	SPF Rats (AITHM Facility) (n = 2)	Laboratory Acclimated SPF Rats (n = 2)	Conventional Rats (Bush House) (n = 2)
*Bordetella bronchiseptica*	Culture	0/2	0/2	0/2
*Bordetella spp*	Culture	0/2	0/2	0/2
*Corynebacterium kutscheri*	Culture	0/2	0/2	0/2
*Klebsiella oxytoca*	Culture	0/2	0/2	0/2
^#^ *Klebsiella pneumoniae*	Culture	0/2	1/2^#^	0/2
*Other significant organisms*	Culture	0/2	0/2	0/2
*Pasteurella pneumotropica*	Culture	0/2	0/2	0/2
*Pasteurella spp*	Culture	0/2	0/2	0/2
^^^ *Proteus spp*	Culture	2/2^	2/2^	0/2
*Pseudomonas aeruginosa*	Culture	0/2	0/2	0/2
*Salmonella spp*	Culture	0/2	0/2	0/2
*Staphylococcus aureus*	Culture	1/2	0/2	0/2
*Streptococcus pneumoniae* (alpha haem)	Culture	0/2	0/2	0/2
*Streptococcus spp* (beta haem)	Culture	0/2	0/2	0/2
**Ectoparasite**				
*Mycoptes musculinis*	Exam	0/2	0/2	0/2
*Myobia musculi*	Exam	0/2	0/2	0/2
*Polyplax spinulosa*	Exam	0/2	0/2	0/2
*Radfordia spp*	Exam	0/2	0/2	0/2
**Endoparasite**				
*Giardia muris*	Wet prep/Motility	0/2	0/2	0/2
*Pinworm – Aspiculuris spp*	Wet prep/Motility	0/2	0/2	0/2
*Pinworm – Aspiculuris tetraptera*	PCR	0/2	0/2	0/2
*Pinworm – Syphacia muris*	PCR	0/2	0/2	0/2
*Pinworm – Syphacia spp*	Wet prep/Motility	0/2	0/2	0/2
*Spironucleus muris*	Wet prep/Motility	0/2	0/2	0/2
**Non-Pathogenic Protozoa**				
*Chilomastix bettencourti*	Wet prep/Motility	0/2	0/2	0/2
*Entamoeba muris	Wet prep/Motility	1/2*	1/2*	1/2*
*Tritrichomonas muris*	Wet prep/Motility	0/2	0/2	0/2

Microbiology results obtained from Cerberus Sciences on two SPF animals, two SPF animals acclimated for seven days in a conventional facility, and two conventional animals. Results presented as number of positive samples per number tested (2).

^#^*Klebsiella pneumoniae* has been associated with bacteraemic disease and widespread abscesses. They inhabit the gastrointestinal tract and are opportunistic agents waiting for the correct conditions to cause infections.

^^^*Proteus species* are gram-negative, rod-shaped, and facultatively anaerobes. They are ubiquitous in the environment and can be cultured from the upper respiratory tract and feces. They are commonly the cause of urinary tract infections in immunocompromised animals. The most common species is *Proteus mirabilis*.

^*^Entamoeba muris are considered non-pathogenic and frequently found in intestinal contents of normal rodents.

## Discussion

Choosing an appropriate animal model for basic research and preclinical studies for translation is extremely complex^1,13,14^. In the process of shifting animal housing and breeding from a conventional to a new SPF facility at the AITHM, James Cook University, we were unable to reproduce basic baseline physiological data that have been routine in our laboratory for over 10 years, and compatible with published data from other laboratories. We report that SPF animals were hemodynamically-compromised, arrhythmogenic with prolonged QTc, coagulopathic, and had significantly higher white blood cell, lymphocyte and monocyte counts, and lower hemoglobin, compared to conventional rats. This new SPF-phenotype led to a lower physiological reserve in response to anesthesia and surgical stress.

Following isoflurane anesthesia and femoral artery and vein cannulation, arterial blood pressure in SPF animals had a decreased waveform accompanying a significant 38% fall in pulse pressure compared to conventional rats (*p* < 0.05) (Fig. 1a,b). Since pulse pressure depends largely on stroke volume and arterial stiffness^15^, our data suggest that the significantly lower pulse pressure in SPF rats was the result of a lower stroke volume and impaired left ventricular-arterial coupling relationship. This was further supported by a prolonged QTc interval in SPF animals (Table 1). A prolonged QTc in animal models and humans have been associated with lower stroke volume, compromised left ventricular function and increased mortality^16,17^. Our interpretation of a failing cardiovascular system in SPF animals was further supported by the presence of ventricular arrhythmias (Table 1, Fig. 2a,b), and a mild cardiomyopathy indicated from a histopathological multifocal myofiber degeneration, replacement fibrosis and macrophage infiltration (data not shown). Blood pressure and heart rate oscillations (timings of peaks and valleys) were similar between SPF and conventional animals (Fig. 1a,b), indicating there was little or no differences in the baroreflex input contributions to blood pressure regulation in the two colonies^18^.

Assessment of coagulation properties also revealed the presence of a significant whole blood hyperfibrinolysis in SPF compared to conventional rats. Hyperfibrinolysis is a common bleeding phenotype following severe traumatic injury, shock or major surgery, and believed to be initiated by activation of protein C on the endothelium-thrombomodulin-thrombin complex^19–21^. However, in our study, SPF animals showed hyperfibrinolysis after minor surgical cut-downs and femoral artery cannulations and instrumentation, a time when baseline data is normally collected prior to experiment. Based on hemodynamic and coagulation status, we conclude that SPF animals possessed a lower physiological reserve to stress of surgery than conventional rats.

The 1.4- to 2.7-fold higher baseline counts of white blood cells, monocytes, lymphocytes, neutrophils and platelet counts, and lower hemoglobin levels, compared to the blood of conventional rats are suggestive of an activated immune system and/or low-grade systemic inflammation. However, when we reverted back to using animals from the conventional facility, a normal hematological profile was restored. Our study supports the work of Beura and colleagues who reported that their ‘standard’ SPF mice were infection-prone as a result of an immature immune system, which was reversed when wild mice were introduced into the colony^6^. They traced the anomaly to alterations in the animal’s gut microbiome.

Although we do not know the reasons for the abnormal hematology in SPF rats, independent external assessment revealed the exclusion of a number of infectious agents (Table 3), with the exception of *Proteus* spp. bacteria. Like other gram-negative bacteria, *Proteus* can release lipopolysaccharide endotoxins, and other reactive substances (e.g. thiobarbituric acid, superoxide radicals) that can translocate across the gut wall and trigger a host’s inflammatory response and possible autoinfection^22^. Thus, the presence of *Proteus* bacteria may have contributed to the SPF rats’ hematological profile and reduced stress response. However, we cannot exclude the possibility of effects of other infectious agents that were not investigated (see Table 3). *Proteus* may have increased in numbers in our SPF colony from the selected exclusion of pathogens in Table 3 that may normally keep *Proteus* spp. numbers in check^22^.

This study was an observational sequential study, with limited animal numbers for some of the outcome measures. Despite these limitations, our findings have a number of important implications for basic, goal-directed and translational research involving animals. Given the influence of the microbiome on the host’s physiology such as altering the immune system, the gut-brain axis, circadian rhythm and the cardiovascular system^23,24^, slight differences have the potential to significantly alter experimental outcomes. This consideration of the gut microbiome on data reproducibility (and replicability) in animal models of disease and injury have been largely ignored or grossly under-reported in the literature^25^. Pathogen status may be an unwanted variable and contribute to subtle differences in experimental outcomes within a single laboratory or between laboratories around the world^10^, and a factor in the failure to translate new therapies from animal studies to humans^26^. On the other hand, the gut microbiome could be manipulated and used as a tool to investigate specific diseases in animal models within defined boundaries^1^. In an attempt to address this variability in animal studies, we propose the following recommendation, which could accompany a *Data Availability Statement* requested by many journals. At the end of a scientific publication involving animals, the author(s) could be mandated to write the following^1^.

### Animal pathogen status availability

A list of infectious agents in animals supporting the conclusions of this study are available by contacting the author(s) and/or institutional data hub (with an appropriate URL).

We conclude that SPF animals displayed an abnormal hemodynamic, hematological and bleeding phenotype in response to anesthesia and minor surgery. Returning to a conventional facility restored normal host physiology. Our results highlight the importance of understanding the effect of infectious agents present (and excluded) in SPF animals on experimental design and research outcomes. Further studies are warranted examining gut microbiome differences, and their implications, to basic and translational research.

## Materials and Methods

### Ethical considerations

This study conforms to the National Health and Medical Research Council Australian Code for the Care and Use of Animals for Scientific Purposes, 8^th^ Edition, 2013, and the Guide for Care and Use of Laboratory Animals published by the US National Institutes of Health (8^th^ Edition, revised 2011), and complies with the Queensland Animal Care and Protection Act, 2001 (Act No.64 of 2001) and James Cook University guidelines. The study was approved by James Cook University Animal Ethics Committee (IACUC approval #A2296) and US Animal Care and Review Use Office (ACURO).

### Conventional sprague dawley colony

Male Sprague-Dawley rats (300–400 g) bred in the conventional James Cook University Breeding Colony were housed in open top caging in a 14–10 hr light-dark cycle with free access to standard rat chow (Norco rural stock) and water *ad libitum*. Cages were polypropylene with high-top wire mesh cage tops (1640 cm^2^ floor space). Bedding was Andersons Bed-o’Cobs® ¼” and large Aspen chew blocks (40 × 40 × 200 mm), PVC tubing, and shredded paper were provided for environmental enrichment. The conventional facility has no barrier or containment restrictions, and the rats were used for a traumatic hemorrhage study under the same ethics approval number. These experiments were carried out before the transition to SPF animals.

### SPF sprague dawley colony

Male Sprague-Dawley rats (300–400 g) bred in the specific-pathogen free (SPF) Australian Institute of Tropical Health and Medicine (AITHM) Small Animal Facility were maintained in individually ventilated double decker Tecniplast cages (1862 cm^2^ floor space) in a 14–10 hr light-dark cycle and fed irradiated rat cubes from Speciality Feeds (Glen Forrest, Western Australia). Nutritional content of feed for both colonies was equivalent and exceeded National Research Council (NRC) guidelines. All bedding and materials for environmental enrichment (shredded paper, tubes, chew blocks, tissues) and for nesting were identical to those used in the Conventional colony with the exception of being autoclaved before use. The AITHM Small Animal Facility has strict containment procedures.

### Animal acclimation

Seven days prior to surgery, animals from both facilities were transferred to the Medical Research Laboratory and housed in individually ventilated double decker Tecniplast cages containing non-sterilised Andersons Bed-o’Cobs® ¼” corncob bedding with Aspen chew blocks, tunnels and shredded paper to allow for enrichment and natural behaviours.

### Animal preparation and instrumentation

Animals were anesthetised in a 2 L induction chamber (9.5 × 22.3 × 9.5 cm) with isoflurane 5% (in 100% oxygen) during the induction phase and 2.5% during any surgical instrumentation, with animals breathing spontaneously. Animals were placed supine in a custom-designed cradle with 2.5% isoflurane and 100% oxygen supplied via nose cone. Aseptic techniques were used for surgery and all surgical equipment was autoclaved prior to use. Sterile chronic catheters (Access Technologies, USA) were inserted in the left femoral artery and vein of anesthetised animals for blood sampling and blood pressure monitoring^20^. Animals were euthanized using pentobarbitone sodium, 325 mg/ml (Lethabarb (100 mg/kg) injectable.

### Blood pressure (n = 5 SPF animals, n = 5 conventional animals)

The femoral artery catheter was connected to a pressure transducer and Bridge Amplifier/Powerlab (AD Instruments) for blood pressure (BP) monitoring. In contrast to hemotology (n = 34, see below), only 5 SPF animals were used for blood pressure analysis and 20 for electrocardiogram (ECG) measurements (see below), because the study was terminated after the adverse events were reported to the Institutional Animal Welfare Officer and Animal Ethics Committee. Blood pressure and ECG data were compared to the hemodynamics of conventional animals measured just prior to the SPF animal experiments.

### Electrocardiograph (ECG) (n = 20 SPF animals, n = 20 conventional animals)

ECG leads were implanted subcutaneously in a lead II configuration (right and left forepaws and right hind paw) to measure heart rate^20^. Electrocardiograms were analysed using the LabChart Pro ECG Analysis module (AD Instruments) with rat beat detection settings and Bazett correction for QT. Arrhythmias were identified using the Lambeth convention as previously described by Canyon and Dobson^27^. Premature ventricular contractions (PVCs) were defined as discrete and identifiable premature QRS complexes. An episode of bigeminy was recognized as a variant of PVCs and characterized by the minimum sequence: P, QRS, PVC, P, QRS, PVC^28^. Salvos were defined as two or three consecutive PVCs, and ventricular tachycardia (VT) was defined as a run of four or more consecutive ventricular premature beats^28^.

### Hematology (n = 34 SPF animals, n = 100 conventional animals)

Whole blood (0.5 ml) was collected from the femoral artery catheter of SPF animals (n = 34) to measure complete blood counts using the VetScan HM5 analyser (REM Systems, Macquarie Park, New South Wales). The hematology results reported for conventional animals (n = 100) were from a study immediately prior to transitioning to SPF animals.

### Coagulation parameters (n = 2 SPF animals; n = 2 conventional animals)

Rotational thromboelastometry (ROTEM^®^, Tem International, Munich, Germany) analysis was conducted on citrated whole blood samples according to manufacturer’s instructions to measure clotting time (CT; sec), maximum clot firmness (MCF; mm), actual clot firmness 60 min after clot initiation (ACF; mm), and maximum clot lysis (ML; %)^29,30^. Assays included EXTEM (extrinsically-activated test using tissue factor) and APTEM (activation as for EXTEM with aprotonin). Aprotinin at low levels (10–50 KIU/mL) inhibits plasmin activation of fibrinolysis and plasmin-induced platelet activation^31^. Hyperfibrinolysis is defined as ML ≥ 15%^32,33^ and confirmed with APTEM test.

### Health screening, macropathology and histopathology (n = 2 SPF animals; n = 2 conventional animals)

Two male rats from the James Cook University conventional Sprague-Dawley colony, two male SPF rats from the AITHM Small Animal Facility, and two male SPF rats acclimated for seven days in a conventional facility were sent to Cerberus Sciences (Adelaide, South Australia) for investigation by an independent Specialist Veterinary Pathologist. Enteric and respiratory bacteriology and endo- and ecto-parisitology assessments were performed using direct examination, culture, and PCR methods.

### Statistical analysis

SPSS Statistical Package 25 was used for statistical analysis (IBM). All values are expressed as mean ± SEM. Complete blood count, hemodynamic and ECG data were evaluated using one-way analysis of variance (ANOVA). Statistical significance was defined as *P* < 0.05. Post-hoc power analysis was performed using G*Power 3 program.

## Data Availability

The datasets supporting the conclusions of this article can be made available by emailing the corresponding author.
